# Irisin: A Potential Link between Physical Exercise and Metabolism—An Observational Study in Differently Trained Subjects, from Elite Athletes to Sedentary People

**DOI:** 10.1155/2017/1039161

**Published:** 2017-03-13

**Authors:** Stefano Benedini, Elena Dozio, Pietro Luigi Invernizzi, Elena Vianello, Giuseppe Banfi, Ileana Terruzzi, Livio Luzi, Massimiliano Marco Corsi Romanelli

**Affiliations:** ^1^Department of Biomedical Sciences for Health, Università degli Studi di Milano, Via L. Mangiagalli 31, 20133 Milan, Italy; ^2^Endocrinology Unit, IRCCS Policlinico San Donato, Via R. Morandi 30, San Donato Milanese, 20097 Milan, Italy; ^3^Laboratory of Experimental Biochemistry and Molecular Biology, IRCCS Istituto Ortopedico Galeazzi, Milan, Italy; ^4^Vita-Salute San Raffaele University, Milan, Italy; ^5^Diabetes Research Institute, Metabolism, Nutrigenomics and Cellular Differentiation Unit, San Raffaele Scientific Institute, Milan, Italy; ^6^Metabolism Research Center, IRCCS Policlinico San Donato, Via R. Morandi 30, San Donato Milanese, 20097 Milan, Italy; ^7^SMEL-1 Laboratory Medicine Unit, IRCCS Policlinico San Donato, Via R. Morandi 30, San Donato Milanese, 20097 Milan, Italy

## Abstract

We compared irisin levels among groups of differently trained healthy individuals to explore the role of irisin as a physiological linker between exercise and metabolic health. Irisin and biochemical parameters of glucose and lipid metabolism were assessed in 70 healthy volunteers stratified for sport performance level into four groups: (1) 20 elite athletes of national level, (2) 20 subelite athletes of local level, (3) 20 recreational athletes, and (4) 10 sedentary subjects. All biochemical parameters were within the ranges of normality. Fasting glucose, HOMA-IR, and total cholesterol levels were inversely related to the degree of physical activity. HbA1c was higher in elite athletes compared to all the other groups (*p* < 0.01). A U-shaped relation between free fatty acids and the degree of physical activity was observed. All groups showed similar plasma irisin levels. After correction for the degree of insulin resistance (irisin/HOMA-IR), elite athletes showed higher levels compared to sedentary and recreational subjects (*p* < 0.01 and *p* < 0.05, resp.). In addition, the number of metabolic parameters correlated with irisin increased at increasing the training status. Our study suggests a correlation between sport performance, insulin sensitivity, and irisin levels. Irisin may be one potential mediator of the beneficial effects of exercise on metabolic profile.

## 1. Introduction

The role of physical activity in maintaining good health and preventing insulin resistance, type 2 diabetes mellitus (DMT2), obesity, metabolic syndrome, atherosclerosis, and other cardiovascular complications is well recognized [1–3]. The protective effect of physical activity may be ascribed, to some extent, to the release of myokines from contracting skeletal muscles. These molecules, in fact, may mediate the beneficial effects of exercise on glucose and lipid metabolism and on inflammation, which characterizes both metabolic and cardiovascular diseases [4]. An interesting editorial by Polyzos and colleagues highlighted the possibility that irisin in humans could be a link between physical activity and metabolic homeostasis and could be implicated in processes involved in weight balance [5].

Irisin is a myokine firstly identified for its ability to induce browning of white adipose tissue, to increase energy expenditure and to protect against insulin resistance and obesity [6]. The beneficial effects of exercise on bone metabolism seem to be mediated by different myokines as irisin [7]. The real effect of physical activity in promoting expression and secretion of irisin in human is still unclear. In fact, besides some studies which described a great effect of exercise in promoting irisin increase [8–10], other studies reported that neither acute nor chronic exercise promotes changes in irisin level [11–14]. Probably, most of these controversies may depend on the type of exercise (resistance/endurance), its duration (acute/prolonged), the training status of individuals before enrollment in the studies, and the type of assay for irisin quantification.

A review published in 2014 explains that different studies in humans have shown inconsistent results related to the proposed induction of irisin by exercise [15]. Human studies till now performed were almost interventional studies aimed to evaluate acute changes in irisin level after a training program. The relationship among the sporting behavior and chronic irisin level is poorly explored, instead.

To this aim, we compared irisin level among groups of differently trained healthy individuals (elite athletes of international and national level, athletes of local level, recreational athletes, and sedentary individuals) and we explored the role of irisin as a physiological linker between exercise and metabolic health.

## 2. Methods

### 2.1. Aim

The purpose of this work is to compare irisin level among groups of differently trained healthy individuals (elite athletes of international and national level, athletes of local level, recreational athletes, and sedentary individuals) and to explore the role of irisin as a physiological linker between exercise and metabolic health.

### 2.2. Design and Setting of the Study

All subjects involved in this protocol were admitted to the UO Endocrinology after an overnight fast and before any exercise activity. At 08.00, a polyethylene catheter was inserted into the antecubital vein of one forearm. At 08.30, a basal blood sample for the measurement of hormones and metabolites was drawn. Weight and height were then recorded with standard scales and stadiometers and BMI was calculated. Then the subjects were discharged.

### 2.3. Biochemical Assays

Blood samples were collected after overnight fasting into pyrogen-free tubes with EDTA as anticoagulant or in tubes for serum separation. Plasma and serum samples were prepared by centrifugation at 4°C within 1.5 h of sampling and aliquots were frozen at −20°C until later analyses.

Fasting glucose, glycated hemoglobin (HbA1c), insulin, total cholesterol, and triglycerides were quantified with commercial kits using Cobas 6000 analyzer (Roche Diagnostics, Milan, Italy), as previously reported [16, 17]. Briefly, total cholesterol has been determined by an enzymatic-colorimetric method. The lower detection limit was 3.86 mg/dL. Intra- and interassay coefficient of variations (CV%) were 1.1% and 1.6%, respectively. The hexokinase method was used to quantify glucose. The lower detection limit was 2 mg/dL. Intra- and interassay CV% were 1.0% and 1.3%, respectively. Turbidimetric inhibition immunoassay method was used for the determination of HbA1c. Lower detection limit was 0.18 mmol/L. Intra- and interassay CV% were 1.6% and 2.0%, respectively. Triglycerides were quantified using an enzymatic-colorimetric method. Lower detection limit was 8.85 mg/dL. Intra- and interassay CV% were 1.1% and 2.0%, respectively. Insulin was measured by electrochemiluminescent immunoassay. The lower detection limit was 0.2 *μ*U/mL. Intra- and interassay CV% were 1.5% and 4.9%, respectively. Free fatty acids (NEFA) were quantified on a Uvikon analyzer using the colorimetric method provided by Randox Laboratories (Crumlin, County Antrim, UK). The method is linear up to 2.24 mmol/L. The minimum detectable level with acceptable precision has been determined at 0.072 mmol/L. Within run assay CV% and total assay CV% were 4.81% and 4.51%, respectively.

### 2.4. Irisin Quantification

Circulating irisin levels have been quantified on serum samples by a specific competitive enzyme immunoassay kit which has been previously validated by MS spectrometry analysis (Cat. EK-067-29, Phoenix Europe GmbH, Karlsruhe, Germany). The minimum detectable concentration was 1.43 ng/mL. The intra- and interassay variations were less than 10% and 15%, respectively. The assays used to detection of irisin were previously validated [18].

### 2.5. HOMA-IR

Insulin action was assessed using the equations provided by HomeOstasis Model Assessment for estimating insulin resistance (HOMA-IR): HOMA-IR = *G*0 · *I*0/22.5, where *I*0 (*μ*U/mL) is the fasting insulin concentration, *G*0 (mmol/L) is the fasting glucose concentration, and 22.5 represents a constant applied to correct the value to unity as previously described [19].

### 2.6. Characteristics of Participant

In a case-control study, we studied 4 groups of healthy subjects who routinely perform different levels of aerobic physical activity (elite athletes of international and national level, athletes of local level, recreational athletes, and sedentary subjects). Elite athlete is defined as a highly specialized athlete whose performances correspond to the best national results in his or her respective sports or discipline. Athlete of local level is a subject who trains at least 4 times a week for a total of at least 10 hours per week. Subjects who performed recreational physical activity are defined as subjects who train at least 2 times a week for a total of at least 3 hours per week. The sedentary is a subject who does not perform any train session in the week and does not perform physical exercise during habitual work.

All the subjects recruited for the study gave their informed written consent after being given an explanation of purposes and nature of the experimental protocol, conducted in accordance with the declaration of Helsinki, as revised in 2013.

### 2.7. Ethics Approval and Consent to Participate

The study was approved by the ethics committee of Ospedale San Raffaele, reference number: 88/int/2016. All the subjects recruited for the study gave their informed written consent after being given an explanation of purposes and nature of the experimental protocol. All procedures performed in studies involving human participants were in accordance with the ethical standards of the institutional and/or national research committee and with the 1964 Helsinki declaration and its later amendments or comparable ethical standards.

### 2.8. Statistical Analysis

All statistical analyses and graphical data representations were done using GraphPad Prism 5.0 biochemical statistical package (GraphPad Software, San Diego, CA). Data are expressed as mean ± SD. The Kolmogorov-Smirnoff test was used to assess the normality of data distribution. Comparisons between groups were performed using ANOVA or Kruskal-Wallis tests followed by Bonferroni or Dunn's posttests, as appropriate. The univariate associations between irisin, demographic, anthropometric, and biochemical parameters were examined by Pearson or Spearman correlation tests. A *p* value < 0.05 was considered significant.

## 3. Results

### 3.1. Anthropometric Parameters of the Participants, Group Size, and Gender

Of the 70 subjects enrolled for the study 20 were elite athletes [17 males and 3 females, age 22.4 ± 2.8 mean ± standard deviation (SD), and body mass index (BMI) 22.4 ± 1.7 Kg/m2], 20 were athletes of local level [15 males and 5 females, age 21.2 ± 2.1 mean ± SD, BMI 22.4 ± 2.4 Kg/m2], 20 were recreational [13 males and 7 females, age 24.6 ± 8.1 mean ± SD, BMI, 22.1 ± 2.7 Kg/m2], and 10 were sedentary individuals [6 males and 4 females, age 25.2 ± 8.1 mean ± SD, BMI, 22.8 ± 2.8 Kg/m2]. Total physical activity score of participants was classified as inactive (less than 600 MET minutes per week), recreational (between 600 and 3000 MET minutes per week), subelite (between 3000 and 4000 MET minutes per week), and elite (more than 4000 MET minutes per week) according to recommendations in the International Physical Activity Questionnaire (IPAQ) [20].

### 3.2. Glucose Metabolism

**Figure 1:**
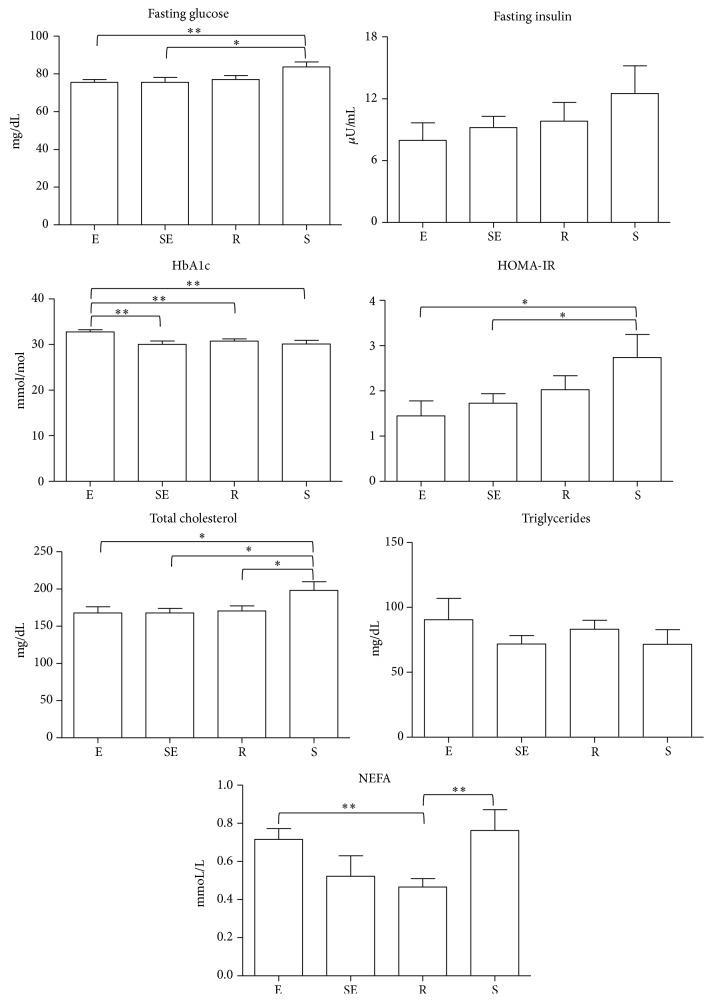
Fasting glucose, insulin, glycated hemoglobin (HbA1c), total cholesterol, triglycerides, free fatty acids (NEFA) levels, and HomeOstasis Model Assessment for estimating insulin resistance (HOMA-IR) values in differently trained subjects. Fasting glucose, insulin, HbA1c, total cholesterol, triglycerides, NEFA levels, and HOMA-IR values were evaluated in elite (E), subelite (SE), recreational (R), and sedentary (S) subjects. ^*∗*^*p* < 0.05; ^*∗∗*^*p* < 0.01.

Fasting glucose, HbA1c, and insulin levels were within the normal ranges in all groups. Fasting glucose was higher in the sedentary group compared to both elite and subelite groups (*p* < 0.01 and *p* < 0.05, resp.) (Figure 1). No statistically significant differences were detected in fasting insulin levels among the groups, although a trend of increase may be observed at decreasing the training status (Figure 1). To be noted, in sedentary, the HOMA-IR was higher compared to both elite and subelite groups (*p* < 0.05 for both), thus suggesting a mild insulin resistance status (Figure 1). HbA1c was increased only in elite athletes compared to all the other groups (*p* < 0.01 for all).

### 3.3. Lipid Profile

Plasma lipid profile was normal in all groups. Total cholesterol was higher in sedentary group than the others (*p* < 0.05, for all). Triglycerides were similar in all the four groups. In contrast, nonesterified fatty acids (NEFA) were higher in sedentary and elite groups compared to recreational (*p* < 0.01 for all) (Figure 1).

### 3.4. Irisin

**Figure 2:**
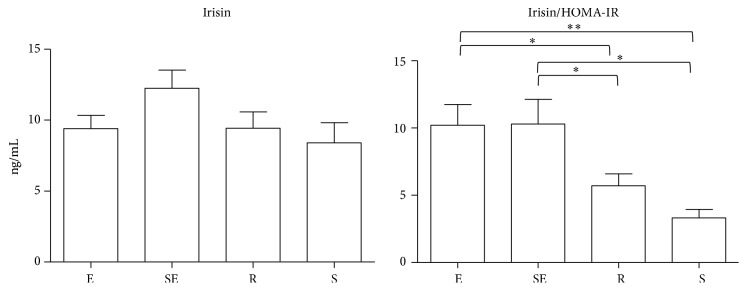
Irisin and irisin/HomeOstasis Model Assessment for estimating insulin resistance (HOMA-IR) ratio levels in differently trained subjects. Irisin and irisin/HOMA-IR ratios were evaluated in elite (E), subelite (SE), recreational (R), and sedentary (S) subjects. ^*∗*^*p* < 0.05; ^*∗∗*^*p* < 0.01.

All groups showed similar plasma irisin level. After irisin correction for the degree of insulin resistance (irisin/HOMA-IR) elite and subelite athletes showed significantly increased ratios compared both to sedentary (*p* < 0.01 for elite and *p* < 0.05 for subelite) and to recreational subjects (*p* < 0.05, for both) (Figure 2). The associations among irisin, demographic, anthropometric, and biochemical parameters were explored using Pearson or Spearman correlation coefficients. For this analysis elite and subelite athletes were considered jointly because these parameters are very similar in these two groups. An increased number of correlations among irisin and biochemical parameters were observed at increasing the training status. In sedentary group, it was only one direct correlation between irisin and irisin/HOMA-IR ratio (Table 1). In recreational group, irisin was also inversely associated with fasting glucose (Table 1). In elite/subelite group, 3 further inverse correlations were observed: with total cholesterol, triglycerides, and free fatty acid (Table 1).

## 4. Discussion

It has been suggested that the myokine irisin could play an endocrine control of metabolism in the humans. Irisin level seems to be negatively correlated with age, insulin concentrations, triglyceride, and adiponectin levels, suggesting that this hormone may be involved in mechanisms for metabolic regulation. In particular in the study of Huh and colleagues an acute bout of vibration exercise increases circulating irisin, whereas chronic training does not change the values of irisin in humans [21]. Another study of this group shows exercise-induced irisin secretion independently of age or fitness level [22]. In our study, we evaluated the concentrations of irisin in a population of athletes who regularly perform different loads of physical activity and we did not find significant differences in the basal concentrations of this hormone in the different groups studied. These data seem to be similar to results of another study performed on adolescent athletes in a paper of Singhal and colleagues [23]. In this study irisin was compared in two groups (eumenorrheic athletes and nonathletes, resp.) in amenorrheic athletes. The data about irisin in the same condition in eumenorrheic athletes and nonathletes were similar in these groups [23]. Another study showed that 12 weeks of heavy strength training is not able to increase serum irisin levels in women [24]. In contrast a study showed, during an endurance exercise routine in sedentary men, a twofold rise in plasma irisin levels at ten weeks [6], while another study in adults reported an increase in irisin following acute exercise, but a decrease in irisin after a 12-week of endurance and strength training [25].

Previous studies focused on insulin resistance [26, 27] assumed that this condition may represent an independent risk factor affecting the circulating levels of different molecules, that is, adipokines, and suggested the use of the adjusted ratio as a more metabolically relevant measure. Since also in our study HOMA-IR is the main parameter that changes more between the different groups, we hypothesized that insulin resistance could affect irisin values and we decided to include also the adjusted ratio (irisin/HOMA-IR) as a new independent parameter. Notably, differently from irisin, whose levels were not different among groups, values of irisin/HOMA-IR were significantly increased when compared to sedentary and recreational subjects, thus confirming a potential influence of insulin resistance on irisin levels.

In our study HOMA-IR correlate inversely with the degree of physical activity (in particular there is a strict correlation between insulin sensitivity and METS expressed as MET minutes per week, *p* < 0.001); logically in subjects that do more physical activities HOMA-IR values are lower and also the values of absolute irisin seem to be high; although not reaching significance this hormone shows a trend of higher values in subjects that perform more physical activity as elite and subelite athletes versus sedentary (10.6 ± 5.1 versus 8.4 ± 4.5 ng/mL). Despite not showing a statistically significant difference these data are interesting and suggest the presence of a “memory” of physical activity performed.

Here are not current data on irisin in elite athletes in literature. The possibility of analyzing irisin on subjects with a high load of exercise (>11 METS: Metabolic Equivalent of Task) is critical to understand the real correlation between this myokine and physical activity.

Another study highlights a significant decrease of irisin in type 2 diabetic subjects compared to nonobese controls [28]; in particular the irisin values found in the lean control subjects are comparable to those found in our group of sedentary subjects.

A recent study showed that irisin concentrations of the endurance trained athletes were higher than that of the sedentary control subjects [29].

At baseline values irisin seems to increase or not different in insulin resistance models such as polycystic ovary syndrome (PCOS) as reported by recent papers [30, 31].

The numerous works in the literature cited in the discussion often have not adequate control group and therefore the findings of these paper must be interpreted and partially downsized in their conclusions.

The values of irisin could be more closely related to insulin sensitivity of peripheral tissues and in particular with striated muscle and this difference seems to be detectable also in basal condition, independently of physical activity performed in acute exercise. In fact, irisin is a myokine produced by striated muscle and the muscle's ability to modulate the input of energy substrates in the muscle is essential to maintain glucose homeostasis.

In fact, inverse correlation between irisin and blood glucose in subjects who performed physical exercises seems to indicate a direct action of irisin on glucose metabolism. In addition high values of HbA1c in elite athletes have already been described in another paper [32] and seem to correlate with increased endogenous glucose production during exercise and secondary to exogenous implementation during and after the performance. The euglycemia that is present in these athletes at rest is probably also mediated by irisin.

Moreover, the presence of lower values of circulating lipids (total cholesterol, triglycerides, and NEFA) in the group of subjects who performed more hours of physical training also indicates a modulatory role of irisin on lipid metabolism.

In particular the possibility of developing a form of “irisin-resistance” linked to the increase in insulin resistance may in part explain the high levels of this hormone in obese subjects and in women with PCOS, while, as already mentioned above, in elite athletes the value of irisin may remain slightly elevated as a “memory” of physical exercise performed in the previous day.

In fact, the absence of changes in irisin values in women with PCOS after taking therapy with metformin suggests, at least in this condition of insulin resistance, a different mechanism of myokine increases [33].

In conclusion our data indicate a possible key role of irisin on glucose and lipid metabolism in health young people, which is reflected on high value of ratio irisin/HOMA-IR in the group of athletes who perform more exercise.

It will need additional studies with a larger number of subjects to better understand the mechanisms that regulate the production of irisin during different levels of physical activity.

## 5. Study Limitations

There are some possible limitations to consider in the present study. First, the number of subjects in the sample is somewhat small. Second, this is a case-control study and the power analysis of sample size cannot draw a cause-effect conclusion about irisin and insulin sensitivity in elite athletes. Further large studies are needed to elucidate the real role of irisin in modulation of insulin sensitivity in humans.

Moreover in some papers cited as references it is not entirely clear whether the blood sample for irisin assay was performed immediately after exercise or after a few hours; this could be a confounding factor that may partially justify the contradictory data.

## Figures and Tables

**Table 1a:** (a) Elite and subelite

	*r*	*p*
Age^(2)^	−0.16	0.336
BMI^(1)^	−0.11	0.512
Fasting glucose^(2)^	−0.477	**0.003**
Fasting insulin^(2)^	−0.058	0.730
Total cholesterol^(1)^	−0.329	**0.047**
Triglycerides^(2)^	−0.441	**0.006**
NEFA^(2)^	−0.545	**0.001**
HbA1c^(1)^	−0.206	0.221
HOMA-IR^(2)^	−0.065	0.698
Irisin/HOMA-IR^(2)^	0.633	**<0.0001**

**Table 1b:** (b) Recreational

	*r*	*p*
Age^(2)^	−0.667	0.967
BMI^(1)^	0.268	0.090
Fasting glucose^(1)^	−0.306	**0.006**
Fasting insulin^(2)^	0.142	0.399
Total cholesterol^(1)^	0.004	0.792
Triglycerides^(2)^	−0.137	0.411
NEFA^(1)^	0.075	0.66
HbA1c^(1)^	−0.139	0.413
HOMA-IR^(2)^	0.203	0.203
Irisin/HOMA-IR^(2)^	0.603	**<0.0001**

**Table 1c:** (c) Sedentary

	*r*	*p*
Age^(2)^	−0.278	0.436
BMI^(1)^	−0.217	0.548
Fasting glucose^(1)^	0.103	0.777
Fasting insulin^(2)^	0.395	0.333
Total cholesterol^(1)^	0.09	0.803
Triglycerides^(2)^	0.4001	0.251
NEFA^(1)^	−0.134	0.752
HbA1c^(1)^	−0.601	0.087
HOMA-IR^(2)^	0.288	0.419
Irisin/HOMA-IR^(2)^	0.833	**0.003**

Associations between variables were explored using Pearson or Spearman correlation coefficients, as appropriate. ^(1)^Pearson correlation; ^(2)^Spearman correlation.

BMI, body mass index; NEFA, free fatty acids; HbA1c, glycated hemoglobin; HOMA-IR, HomeOstasis Model Assessment for estimating insulin resistance.
